# Anesthetic management of airway stent placement by rigid bronchoscopy with superior laryngeal nerve block while preserving spontaneous breathing: A case report

**DOI:** 10.1002/ccr3.8232

**Published:** 2023-11-19

**Authors:** Toshio Okada, Mio Yoshida, Tomoko Matsushita, Yusuke Ishida, Kinya Furukawa, Michihiro Murozono

**Affiliations:** ^1^ Department of Anesthesiology Tokyo Medical University Tokyo Japan; ^2^ Department of Anesthesiology Showa University Hospital Tokyo Japan; ^3^ Department of Thoracic Surgery Tokyo Medical University Ibaraki Medical Center Ibaraki Japan; ^4^ Department of Anesthesiology Tokyo Medical University Ibaraki Medical Center Ibaraki Japan

**Keywords:** air way stent, spontaneous breathing maintenance, superior laryngeal nerve block

## Abstract

**Key Clinical Message:**

The combination of superior laryngeal nerve block can reduce the respiratory depression that occurs during management under total intravenous anesthesia.

**Abstract:**

Anesthetic management of endobronchial stent placement by rigid bronchoscopy requires the maintenance of spontaneous breathing while suppressing upper airway reflexes. The combination of superior laryngeal nerve block (SLNB) can reduce the respiratory depression that occurs during management under total intravenous anesthesia. The patient was diagnosed as having lung cancer with invasion into the right middle bronchus and stenosis of the right main bronchus on chest computed tomography, and emergency airway stent placement was performed. Sedation was initiated with propofol and dexmedetomidine, and ultrasound‐guided SLNB was performed after local anesthetic spraying into the oral cavity and trachea. Bucking was minimally controlled during insertion of the rigid bronchoscope. The patient's intraoperative hemodynamics remained stable, and there were no hypoxic events. SLNB can provide the suppression of the upper airway reflex while minimizing effects on spontaneous breathing, and may be useful for achieving balanced anesthesia during rigid bronchoscopy.

## Graphical abstract

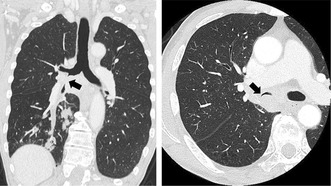

## 1 BACKGROUND

Tracheobronchial stenting is a type of interventional respiratory therapy. In anesthesia management for rigid bronchoscopy, it is necessary to maintain an appropriate depth of anesthesia while maintaining spontaneous breathing. The usefulness of superior laryngeal nerve block (SLNB) in awake intubation is well known.^1^ Recently, the usefulness of SLNB in airway stent insertion has been recognized, but there are few reports of ultrasound‐guided SLNB. In a previous report, the combination of SLNB and total intravenous anesthesia (TIVA) reduced the respiratory depression associated with the use of propofol and fentanyl in anesthesia for rigid bronchoscopy.^2^ Ultrasound‐guided SLNB may be useful for reducing the dose of intravenous anesthetics, by reducing airway reflexes. We here report a case of a patient with airway stenosis caused by lung cancer, who underwent tracheal stent placement under rigid bronchoscopy. TIVA combined with ultrasound‐guided SLNB enabled safe anesthetic management without any adverse events. We obtained written informed consent from the patient to publish this case report.

## 2 CASE PRESENTATION

**FIGURE 1:**
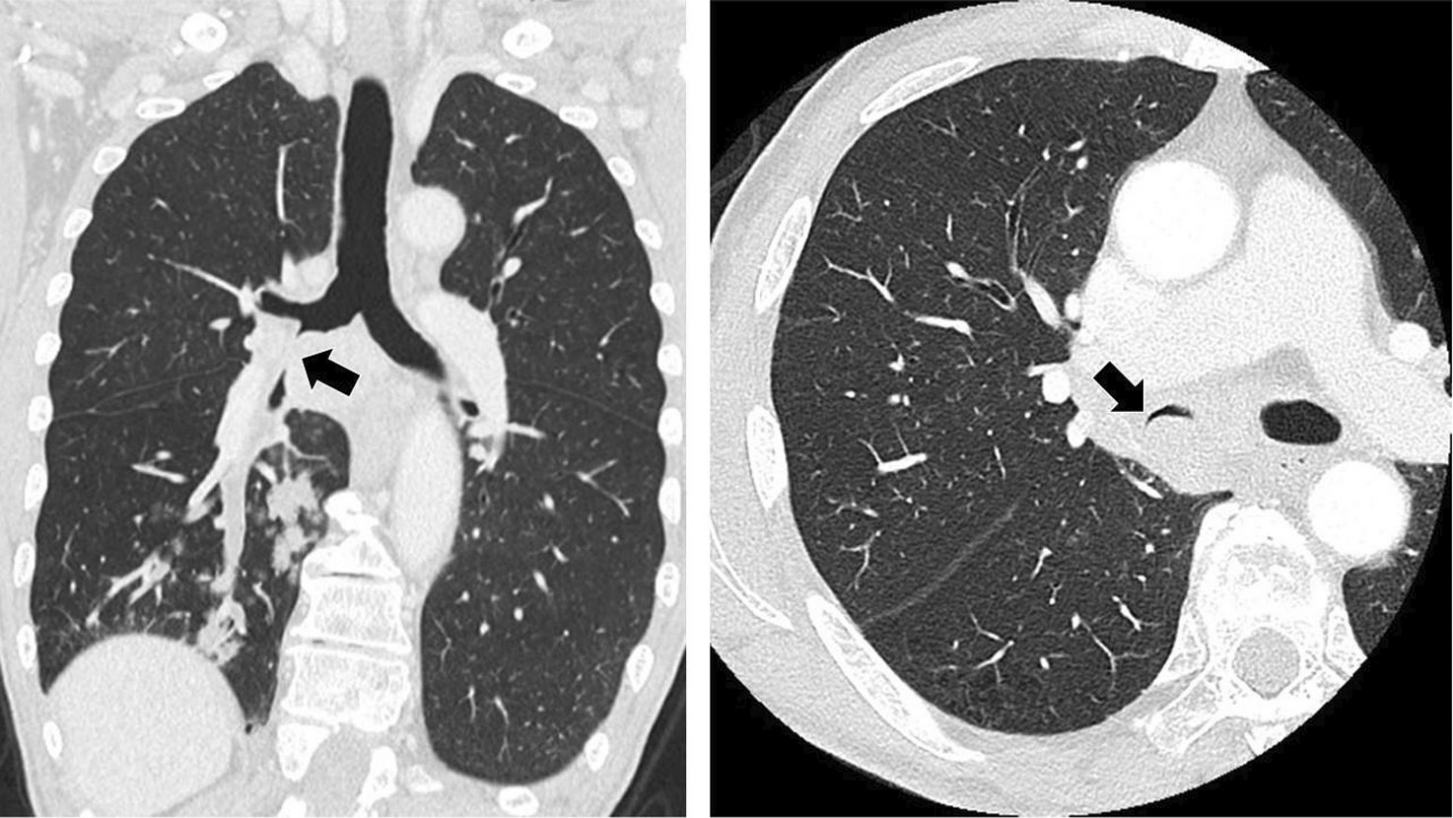
Chest computed tomography on admission. Each image displays a mass lesion in the right lower lung field and enlarged lymph nodes below the tracheal bifurcation. The arrow indicates stenosis of the right middle bronchus.

A 74‐year‐old man presented with a cough persisting for 2 months and weight loss. A chest computed tomography scan displayed a 45‐mm‐long mass lesion in the right lung at the end of S^9^–S^10^, enlarged lymph nodes (35 mm) below the tracheal bifurcation, and infiltration in the right middle bronchus. The right main bronchus was stenotic (Figure 1), which could lead to atelectasis, hence the patient was admitted to our hospital for emergency endobronchial stenting.

**FIGURE 2:**
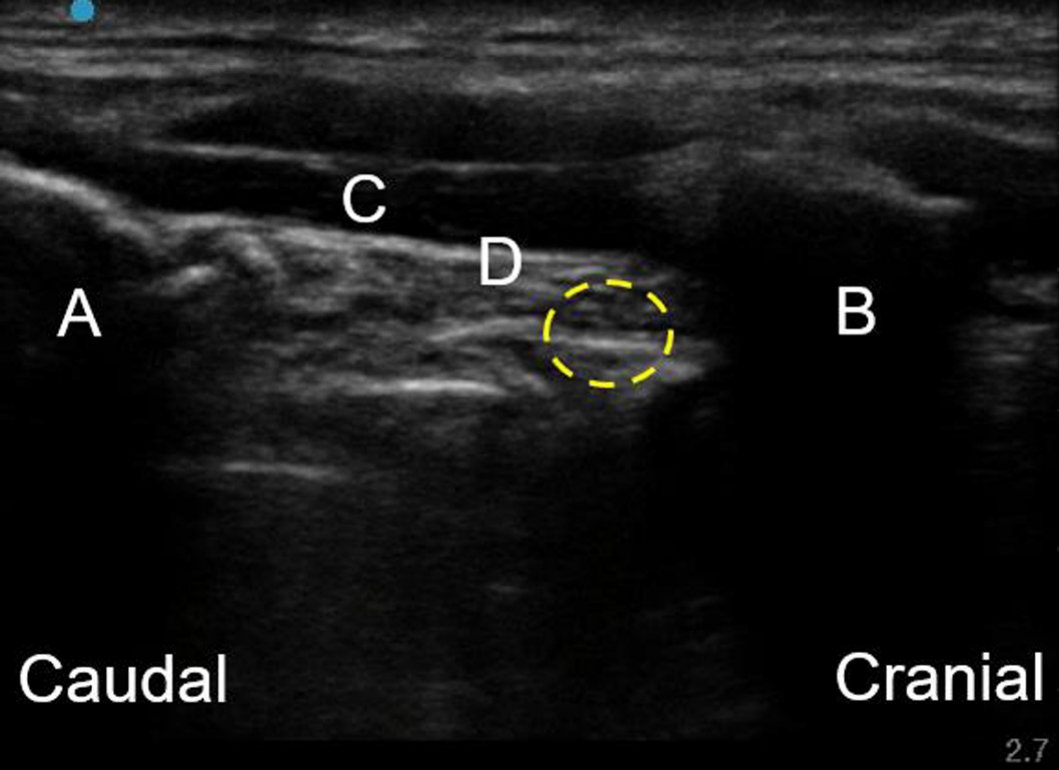
Ultrasound images of structures around the superior laryngeal nerve. Thyroid cartilage (A) hyoid bone, (B) thyrohyoid muscle, (C) and thyrohyoid membrane, (D) are marked on the ultrasound image. The Yellow dotted circle represents the space of the superior laryngeal nerve.

**FIGURE 3:**
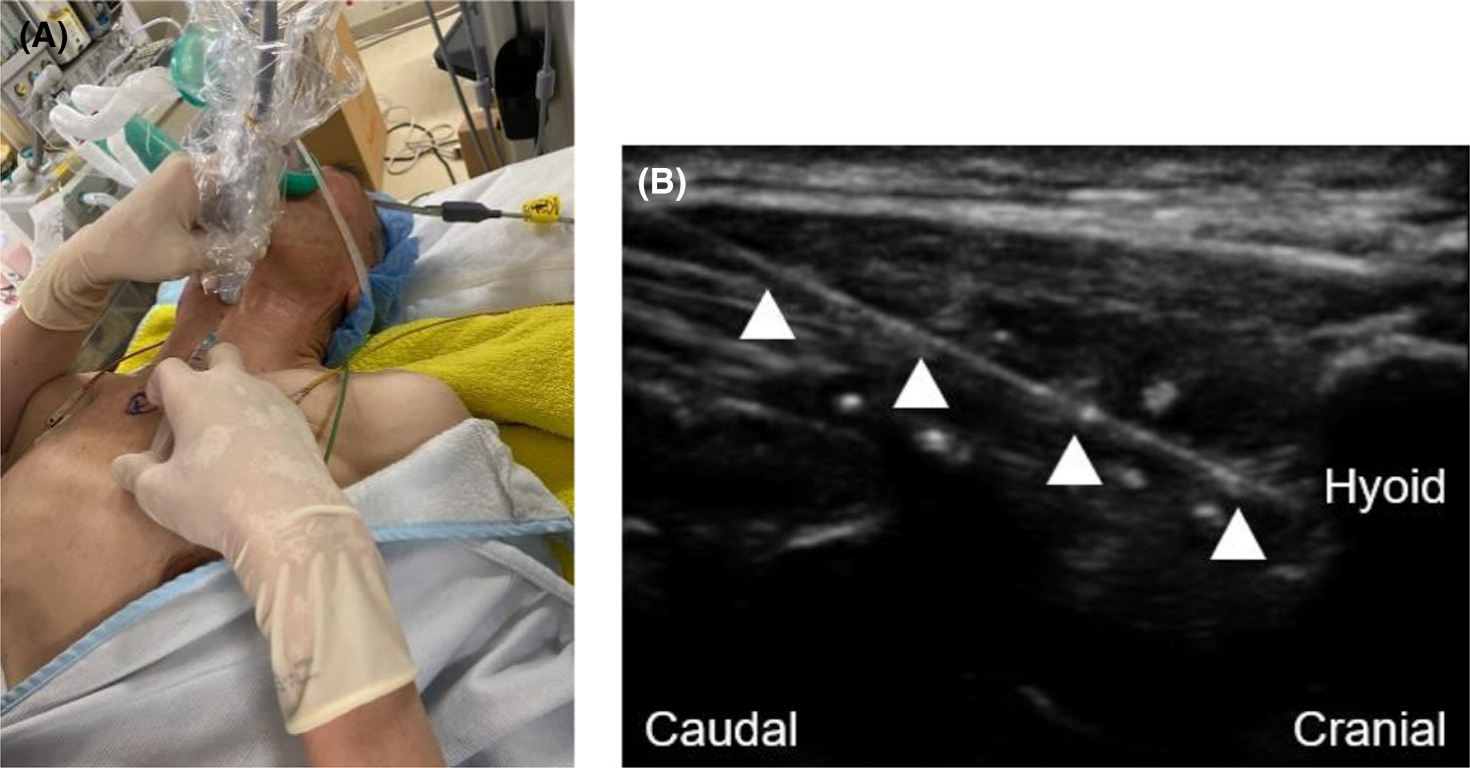
State at the superior laryngeal nerve block. Image of actual implementation of the superior laryngeal nerve block, (A) and a real‐time ultrasound image of the nerve block, (B) The superior laryngeal nerve block was performed in the sagittal plane under real‐time ultrasound guidance. The tip of the needle (indicated by the arrowheads) was positioned around the greater horn of the hyoid bone.

After entering the operating theater, the patient's vital signs were recorded as follows: oxygen saturation, 99% under room air; blood pressure, 176/82 mmHg; and heart rate, 62 bpm. In the supine position, the patient had no respiratory distress. Oxygenation (6 L/min) was started using an oxygen mask. Anesthesia was induced by TIVA combined with propofol and dexmedetomidine. Propofol was administered by target‐controlled infusion (TCI) with a Terufusion® Syringe Pump (Terumo Co., Tokyo, Japan). Dexmedetomidine administration was started at 0.6 μg/kg/h. After intravenous infusion of 50 μg of fentanyl, 5 mL, and 2.5 mL of 4% lidocaine were sprayed into the space between the pharyngeal wall and the glottis and into the trachea, respectively, using a McGrath MAC® video laryngoscope (Medtronic Co., Minneapolis, MN, USA). A pillow was placed under the patient's shoulder to maintain neck extension, and the linear probe (13–6 MHz) Sonosite SII (Fujifilm Sonosite, Inc., Bothell, WA, USA) was placed in the parasagittal plane of larynx to identify the thyroid cartilage, thyrohyoid muscle, thyrohyoid membrane, and hyoid bone (Figure 2). Next, a hockey stick‐type probe (13–6 MHz) was used to identify the greater horn of the hyoid bone, and then SLNB was performed using a 23‐gauge, 60‐mm needle, and a 5‐mL syringe in the sagittal approach under real‐time ultrasound guidance (Figure 3A, B). Lidocaine (2 mL of a 2% solution) was injected to each side of larynx, and no bleeding was confirmed after compression for 5 min after the SLNB. After confirming the anesthetic procedure, the bispectral value remained about 60 and spontaneous breathing was maintained, a rigid bronchoscope was inserted by a respiratory surgeon. There was almost no bucking during insertion. Spontaneous breathing was maintained throughout the procedure, and pure oxygen was supplied through the side port of the rigid bronchoscope. The stenosis was balloon dilated and a 9‐mm silicone stent was inserted. There was little body movement during insertion of the rigid speculum alone, but when endotracheal manipulation was performed, bucking was observed, so a total of 3 mL of 2% lidocaine was intermittently administered into the trachea. Intraoperative hemodynamics remained stable throughout the operation, even though no additional fentanyl was administered. The endoscopic procedure was completed without any adverse events, and the rigid bronchoscope was removed. SpO_2_ was maintained at 97%–100% during the anesthesia. The operation time was 1 h 55 min. There were no complications such as tracheal bleeding or vocal cord paralysis after the surgery.

## 3 DISCUSSION

An airway stent is an artificial endobronchial prosthesis that expands and supports the lumen of the trachea and bronchi, or closes a fistula, in patients with central airway stenosis. There are two types of airway stents currently available in Japan, that is, silicone stents and self‐expandable metallic stents,^3^ and silicone stent implantation is widely accepted as requiring rigid bronchoscopy under general anesthesia.^4^

In general, anesthesia management for rigid bronchoscopy is performed by the following two methods: one is to maintain the patient's spontaneous breathing, and the other is to administer muscle relaxants and perform controlled breathing. In high‐risk patients, such as those with bronchopleural fistulas, treatment with extracorporeal membrane oxygenation has been reported.^5^ Although there is a study report that the incidence of hypoxic events was lower in the controlled breathing group,^6^ as the silicon cap of the rigid scope head is removed during the procedure, this may cause apnea in patients undergoing controlled ventilation. Therefore, at our institution, anesthesia is managed in most patients while maintaining spontaneous breathing.

In a report of TIVA assisted by ultrasound‐guided SLNB and lidocaine endotracheal spray for rigid bronchoscopy, stable spontaneous breathing was maintained during operation without laryngospasm, bucking, or desaturation, and no perioperative pulmonary or cardiovascular complications were observed.^2^ SLNB is often used in awake intubation, similar to rigid bronchoscopy. SLNB in awake fiber intubation is more advantageous than the spray method alone, in terms of the time required for intubation and patient satisfaction.^7^,^8^ In the case of awake fiber intubation, similar results were obtained in patients treated with SLNB and patients treated with continuous remifentanil alone.^9^ SLNB has also been applied clinically for the treatment of neurogenic cough.^10^ In our institution, SLNB is performed when the following criteria are met: (1) the patient does not complain of respiratory distress in the supine position, (2) there is no anatomical abnormality in the appearance of the patient's neck, and (3) the preoperative ultrasound pre‐scan shows a typical image.

The superior laryngeal nerve is a branch of the vagus nerve, and its external branch controls cricothyroid muscle movement, and the internal branch controls the sensation of the laryngeal mucosa above the level of the vocal cords, including the root of the tongue, and the epiglottis.^8^, ^11^, ^12^ Therefore, preoperative and intraoperative spraying of local anesthetic is necessary for anesthesia to suppress the reflex of the trachea. The superior laryngeal nerve runs posterior to the carotid artery, passing through the lateral side of the hyoid bone, and the medial branch passes just below the greater horn of the hyoid bone, approaching the thyrohyoid muscle with the superior laryngeal artery,^13^, ^14^ and then penetrates the thyrohyoid membrane.^12^, ^15^, ^16^ Autopsy results have demonstrated that there is a space between the hyoid bone and the thyroid cartilage, which contains the internal branch of the superior laryngeal nerve.^17^ By placing a linear ultrasound probe in the parasagittal plane under the jaw, the greater horn of the hyoid bone, thyroid cartilage, thyrohyoid membrane, and thyrohyoid muscle can be identified, and the internal branch of the superior laryngeal nerve is detected between the hyoid bone and thyroid cartilage.^12^, ^18^, ^19^, ^20^ Reported complications of SLNB that was performed blindly include unilateral visual impairment with left facial numbness and hearing loss.^21^ Therefore, SLNB performed under real‐time ultrasound guidance is preferable.

When performing rigid bronchoscopy operation at our institution, we start anesthesia with dexmedetomidine (0.5–0.7 μg/kg/h) and propofol by TCI (1.0–2.0 μg/mL) to maintain a sedation level that maintains spontaneous breathing. In recent years, dexmedetomidine, which is a central α_2_‐adrenoceptor agonist, has increasingly been used in postoperative intensive care units for sedation and analgesia.^3^ Dexmedetomidine has also been shown to be useful as a sedative agent that does not cause respiratory depression,^22^ and for maintaining sedation during intubation.^23^ It acts on part of the circuitry that governs natural sleep, and is similar to normal physiological sleep.^24^ Propofol is equally effective, but the risk of airway obstruction and hypoxic events is higher than for dexmedetomidine.^25^

Regarding anesthesia for rigid bronchoscopy, a combination of TIVA and SLNB might be enable avoiding the use of opioids and propofol, and reduce the risk of respiratory depression, but further studies are needed to confirm its usefulness. The location of the superior laryngeal nerve can easily be visualized by ultrasound, and the procedure is simple. Therefore, the combination of TIVA and SLNB may be useful for achieving balanced anesthesia for rigid bronchoscopy.

## AUTHOR CONTRIBUTIONS

**Toshio Okada:** Conceptualization; writing – original draft; writing – review and editing. **Mio Yoshida:** Conceptualization; writing – review and editing. **Tomoko Matsushita:** Conceptualization; writing – review and editing. **Yusuke Ishida:** Conceptualization; writing – review and editing. **Kinya Furukawa:** Conceptualization; writing – review and editing. **Michihiro Murozono:** Conceptualization; writing – original draft; writing – review and editing.

## FUNDING INFORMATION

None.

## CONFLICT OF INTEREST STATEMENT

The authors declare that they have no competing interests associated with this manuscript.

## ETHICS STATEMENT

Not applicable.

## CONSENT

Written informed consent was obtained from the patient for publication of this case report and the accompanying images.

## Data Availability

The data that support the findings of this study are available from the corresponding author upon reasonable request.
